# Seagrass meadows (*Posidonia oceanica*) distribution and trajectories of change

**DOI:** 10.1038/srep12505

**Published:** 2015-07-28

**Authors:** Luca Telesca, Andrea Belluscio, Alessandro Criscoli, Giandomenico Ardizzone, Eugenia T. Apostolaki, Simonetta Fraschetti, Michele Gristina, Leyla Knittweis, Corinne S. Martin, Gérard Pergent, Adriana Alagna, Fabio Badalamenti, Germana Garofalo, Vasilis Gerakaris, Marie Louise Pace, Christine Pergent-Martini, Maria Salomidi

**Affiliations:** 1 Department of Earth Sciences, University of Cambridge, Downing Street, CB2 3EQ Cambridge, Cambridgeshire, United Kingdom; 2 Department of Environmental Biology, University of Rome “La Sapienza”, 32 Viale dell’Università, 00185 Rome, Italy; 3 Institute of Oceanography, Hellenic Centre for Marine Research, PO box 2214, 71003 Heraklion, Crete, Greece; 4 Laboratory of Marine Biology, Department of Biological and Environmental Science and Technologies, University of Salento – CoNISMa, 73100 Lecce, Italy; 5 CNR-IAMC, Via L. Vaccara 61, 91026 Mazara del Vallo, Italy; 6 Department of Biology, Faculty of Science, University of Malta, MSD 2080 Msida, Malta; 7 United Nations Environment Programme World Conservation Monitoring Centre, 219 Huntingdon Road, CB3 0DL Cambridge, Cambridgeshire, United Kingdom; 8 Faculty of Sciences University of Corsica, Campus Grimaldi BP 52, 20250 Corte, France; 9 CNR-IAMC, Via G. Da Verrazzano 17, 91014 Castellammare del Golfo, Italy; 10 Institute of Oceanography, Hellenic Centre for Marine Research, PO box 712, 19013 Anavyssos, Greece; 11 Department of Fisheries and Aquaculture, Fisheries Resource Unit, Ministry for Sustainable Development, the Environment and Climate Change (MSDEC-FRU), Ghammieri, Marsa MRS 3303 (Malta)

## Abstract

*Posidonia oceanica* meadows are declining at alarming rates due to climate change and human activities. Although *P. oceanica* is considered the most important and well-studied seagrass species of the Mediterranean Sea, to date there has been a limited effort to combine all the spatial information available and provide a complete distribution of meadows across the basin. The aim of this work is to provide a fine-scale assessment of (i) the current and historical known distribution of *P. oceanica*, (ii) the total area of meadows and (iii) the magnitude of regressive phenomena in the last decades. The outcomes showed the current spatial distribution of *P. oceanica*, covering a known area of 1,224,707 ha, and highlighted the lack of relevant data in part of the basin (21,471 linear km of coastline). The estimated regression of meadows amounted to 34% in the last 50 years, showing that this generalised phenomenon had to be mainly ascribed to cumulative effects of multiple local stressors. Our results highlighted the importance of enforcing surveys to assess the status and prioritize areas where cost-effective schemes for threats reduction, capable of reversing present patterns of change and ensuring *P. oceanica* persistence at Mediterranean scale, could be implemented.

Seagrass meadows rank amongst the most valuable coastal ecosystems on Earth in terms of goods and services they provide^1^,^2^. Although their structural and functional roles have been largely understood, seagrasses are declining at alarming rates due to climate change (e.g. warming, ocean acidification), alien species invasion and direct human activities near the coasts (e.g. coastal urban development, fishing activities, aquaculture)^3^,^4^. According to Waycott *et al.*^5^, at least 1.5% of seagrass beds is lost every year and almost 29% of the areal extent of seagrass has disappeared globally since 1879, implying that 1/3 of goods and services they provide has been already lost.

*Posidonia oceanica* (L.) Delile is the most important endemic seagrass species of the Mediterranean Sea^6^ and it can form meadows or beds extending from the surface to 40–45 m depth.

Full recovery of *P. oceanica* meadows is usually considered irreversible in human time-scale, because it is a slow-growing species with a low recovery rate^7^. The management of direct impacts, such as trawling, anchoring, dredging and pipeline refilling, can help recovery and promote resilience, although this can take an extremely long time^8^,^9^. Transplantation of seagrass is often unsuccessful, largely due to the fact that habitats are still too deteriorated to allow planted seagrasses to survive^10^.

Therefore, it is crucial to (i) undertake specific actions to mitigate the threats causing regression and (ii) promote good conservation practices before the seagrasses regress, thereby allowing these habitats to fulfil their key roles in coastal areas.

In the last twenty years, *P. oceanica* has become one of the main targets of the protection and management of the Mediterranean marine environment^11^,^12^. The European Union’s Habitat Directive (92/43/CEE) includes *P. oceanica* beds among priority habitats (Habitat Type 1120: *P. oceanica* beds - *Posidonion oceanicae*). Seagrass meadows also have a dedicated Action Plan within the framework of the Barcelona Convention, under the “Protocol concerning Specially Protected Areas and Biological Diversity in the Mediterranean”. More recently, the Marine Strategy Framework Directive (MFSD) (2008/56/EC) has established a framework according to which each Member States shall take the necessary measures to achieve or maintain “Good Environmental Status” in the marine environment. Angiosperms have been listed as a biological feature in Table 1 of Annex III “Indicative list of characteristics, pressures and impacts” and *P. oceanica* has been selected as representative species of the angiosperm quality elements for the Mediterranean marine environment. Parallel to this, each EU Member State has defined its own method to evaluate the health status of *P. oceanica* meadows according to the Water Framework Directive (2000/60/EC)^13^.

In addition, detailed spatial information on habitat distribution is a prerequisite knowledge for a sustainable use of marine coastal areas^14^. First attempts at mapping *P. oceanica* beds date back to the end of the 19^th^ century^15^, although first maps were produced during the early 1970 s in France^16^ and Italy^17^. The implementation of international agreements (Barcelona Convention) and European legislations (Natura 2000, MSFD) have encouraged mapping and monitoring efforts of *P. oceanica* beds in the majority of European countries^18^.

Recently, acoustic devices (i.e. side scan sonar, multibeam echosounder) and remotely operated vehicles^19^,^20^,^21^ have proved to be powerful tools in seabed mapping, allowing the production of accurate and detailed cartography, especially in the deeper waters, whereas aerial photography has given good results in shallow-water^22^. The development of new computerized tools such as Geographic Information System (GIS) software has facilitated the production of detailed and geo-referenced distribution maps of *P. oceanica* with a higher precision than previous works.

Despite *P. oceanica* being one of the most important and well-studied Mediterranean species, there has been to date a limited effort to combine all the spatial information available and provide a synthesis of the current distribution and the total area of beds. Previous studies have been based on a limited number of works with scattered quantitative data^23^ or limited spatial extent^24^ and presence/absence data at a very low spatial resolution^25^. In addition, such datasets have never been made available online, with some limited exceptions^26^. Furthermore, reliable historical information on the distribution of this habitat is largely lacking or has a low accuracy. Therefore, data on current distribution are scarcely informative of the trajectories of change and patterns of regression, which have been assessed only through a limited amount of information on meadow changes^4^. This represents a strong limitation in providing a baseline of past ecosystem conditions^27^. Thus, setting meaningful reference conditions, that might support regression monitoring and recovery assessment, remains a challenge.

This study, which is part of the European Research project Mediterranean Sensitive Habitats (MediSeH)^28^, combines a fine-scale assessment of the current distribution of *P. oceanica*, together with available historical information collected at Mediterranean scale. The aim of the work is to review the current and past distributions of meadows across the basin in order to identify areas showing trajectories of change. We anticipate that our results will provide essential spatial data to support coordinated and comprehensive actions across the Mediterranean basin.

## Results

### Current distribution

The total known area of *P. oceanica* meadows in the Mediterranean Sea was found to be 1,224,707 ha (12,247 km^2^) (510,715 ha in the western and 713,992 ha in the eastern part of the basin) (see Table 1). The seagrass was found to be present along 11,907 linear km out of a total coastline extending over 46,000 linear km, whereas it was absent from 12,622 linear km. For the remaining 21,471 linear km of coastline, no information on presence or absence was available (additional details are listed in Table 2).

**Figure 1:**
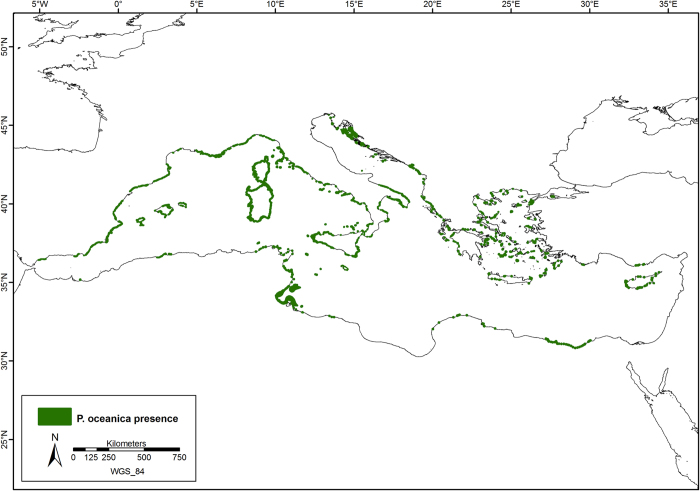
Current distribution of *Posidonia oceanica* meadows. The current distribution of *P. oceanica* (green areas) along the Mediterranean Sea coastline, based on collated spatial information available on meadow presence. Map created with ArcGIS^®^ software by Esri (Environmental Systems Resource Institute, ArcMap 9.3, www.esri.com) using data from OpenStreetMap.org (© OpenStreetMap contributors^59^).

**Figure 2:**
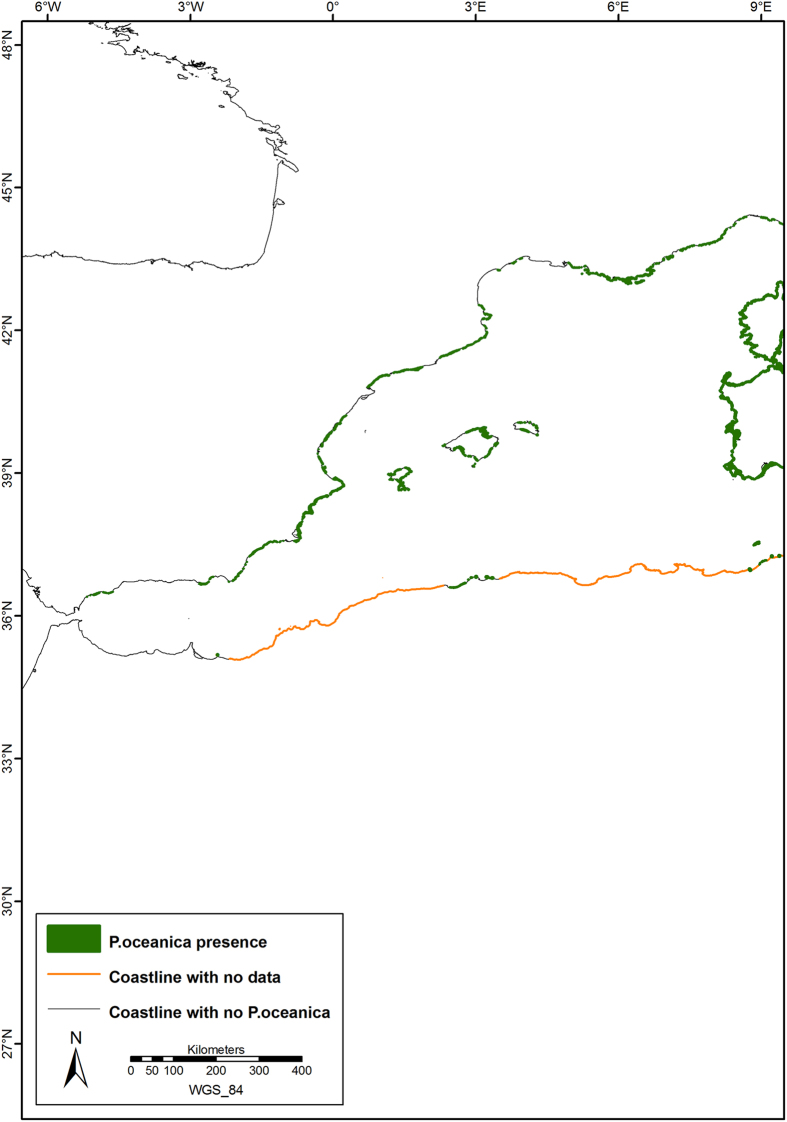
Detail of the current distribution of *Posidonia oceanica* meadows in the Western Mediterranean Sea. Map created with ArcGIS^®^ software by Esri (Environmental Systems Resource Institute, ArcMap 9.3, www.esri.com) using data from OpenStreetMap.org (© OpenStreetMap contributors^59^).

**Figure 3:**
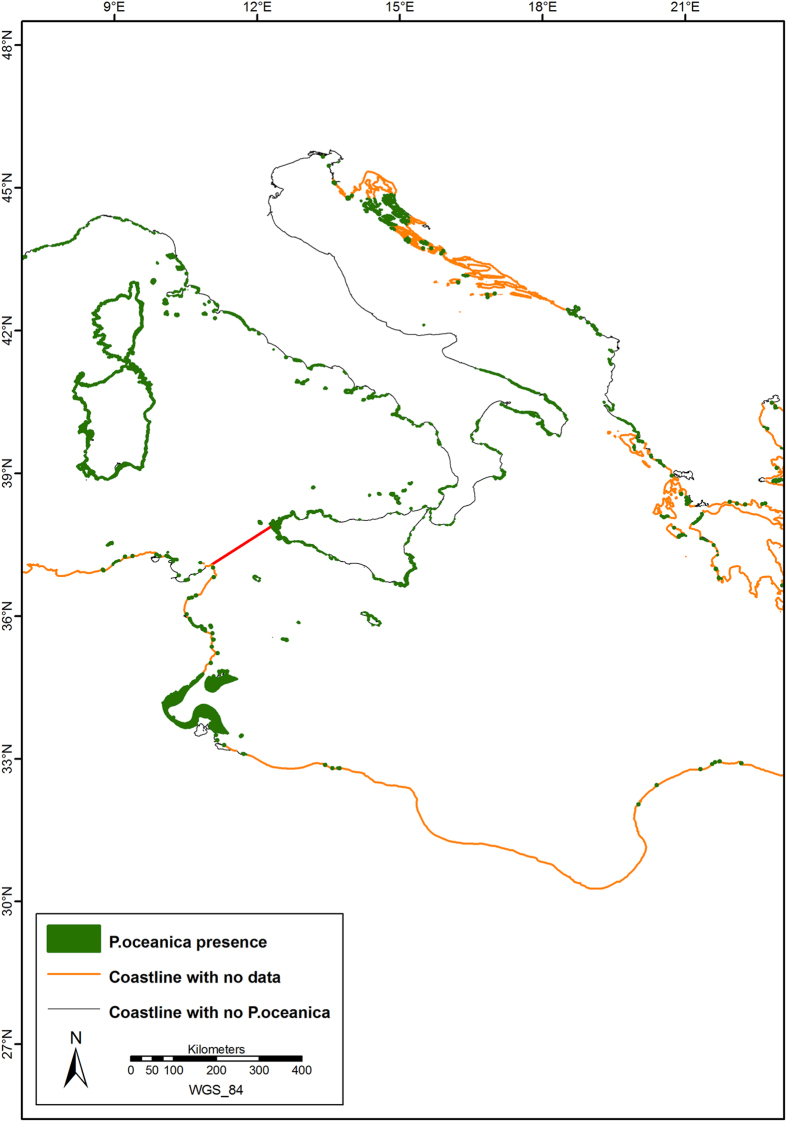
Detail of the current distribution of *Posidonia oceanica* meadows in the Central Mediterranean Sea. The red line marks the border between the Western and the Eastern Mediterranean Basin. Map created with ArcGIS^®^ software by Esri (Environmental Systems Resource Institute, ArcMap 9.3, www.esri.com) using data from OpenStreetMap.org (© OpenStreetMap contributors^59^).

**Figure 4:**
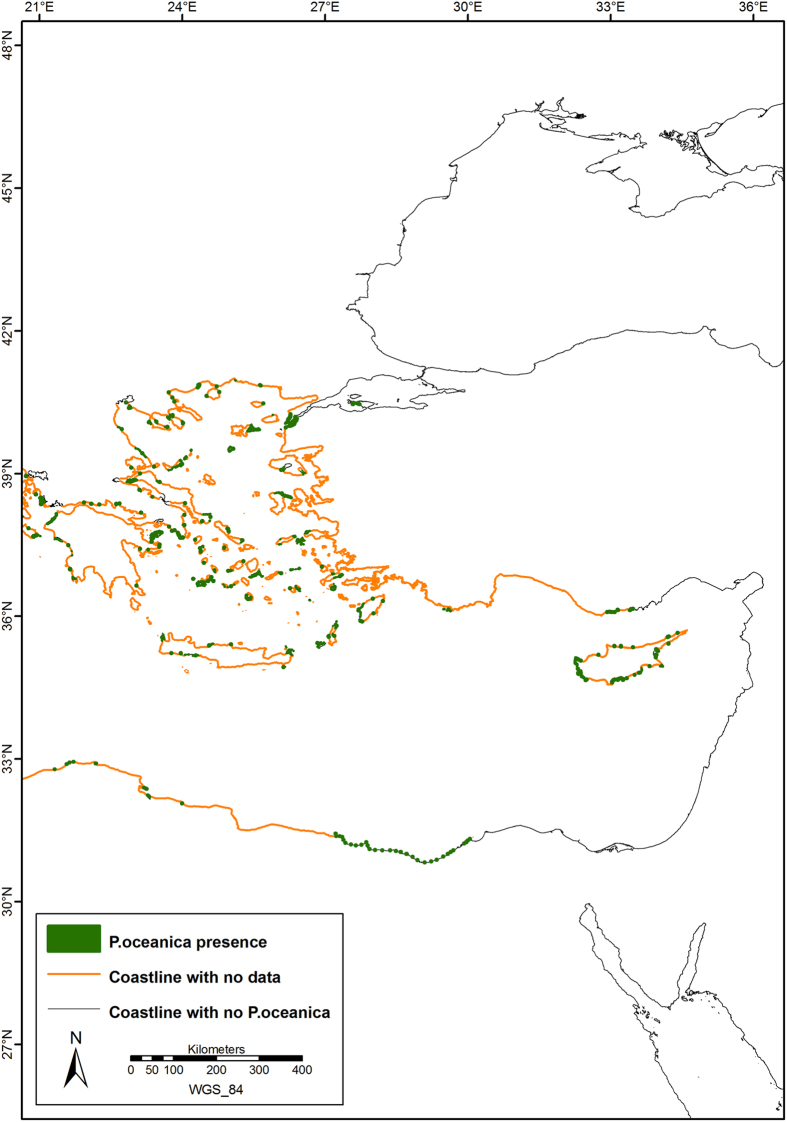
Detail of the current distribution of *Posidonia oceanica* meadows in the Eastern Mediterranean Sea. Map created with ArcGIS^®^ software by Esri (Environmental Systems Resource Institute, ArcMap 9.3, www.esri.com) using data from OpenStreetMap.org (© OpenStreetMap contributors^59^).

The current distribution of *P. oceanica* is shown in Fig. 1, with further details in Figs 2, 3, 4, which show the presence of *P. oceanica* as well as areas where no data exist or where *P. oceanica* is known to be absent.

Knowledge on the distribution of *P. oceanica* meadows was fairly comprehensive in the north-western and central part of the Mediterranean Sea. Cartography was considered complete for the coastline from Spain to Albania, except for parts of Croatia and the southern Mediterranean coasts (from Morocco to Tunisia). Some maps were available from Slovenia to the southern Turkish coastline, but *P. oceanica* was found to be absent in the eastern part of the Mediterranean basin (Syria, Lebanon, Israel and part of the Egyptian coasts, except for the Nile’s delta). Data were available for Malta and some sites along the coasts of the Dardanelles Strait, islands of the Central Marmara Sea and Cyprus. In the southern part of the Mediterranean basin, *P. oceanica* distribution was poorly documented (Algeria and Libya). More detailed information is reported in Table 2 showing the surface area and proportion of coastline with current *P. oceanica* for each country. An extensive and complete list of the collated studies for each Mediterranean region can be found in the Supplementary References and Supplementary Table S1 (see Supplementary Information online).

In Spain, *P. oceanica* meadows were found to be widely present along the continental coastline and islands, with a measured area of 172,699 ha. Along the French coast, *P. oceanica* had a total area of 94,030 ha and it was present, more or less continuously, along the continental coasts and islands (including Corsica). In Italy, *P. oceanica* meadows covered 337,611 ha and it was characterized by a rather continuous distribution along continental and insular coasts of the Tyrrhenian, Ionian Sea, and South-Western Adriatic Sea, with the exception of the main river mouths. Along the North and Central-Western Adriatic coasts, meadows were not present except for a patchy distribution in the northern sector only. In the Eastern Adriatic Sea, small meadows covered 9 ha in Slovenia. *P. oceanica* beds were found along the northern Croatian coasts, with an area of 31,437 ha, whereas “presence points” only were available for the remaining Croatian and the entire Montenegrin coastlines. *P. oceanica* was widely distributed in Albania, where 4,803 ha of meadows were mapped. In the Maltese Islands, *P. oceanica* meadows were concentrated along the north-eastern coastline, covering an estimated area of 5,860 ha. In Cyprus, *P. oceanica* beds were mapped along the entire island’s coastline and available maps showed a total area of 9,040 ha. In Greece, *P. oceanica* was widely present along the majority of continental coasts and was found around the Greek islands covering 44,939 ha, but only a fraction of these meadows have been mapped to date. Along the Turkish coasts, mainly point information about the distribution of *P. oceanica* was available, with a total area of the few mapped meadows amounting to 287 ha. The presence of seagrasses was confirmed along the Turkish Aegean coasts and in few localities along the southern Levantine part of Turkey. A sharp border at 36°09'12” N, 33°26'39” E represented the eastern *P. oceanica* boundary along the continental coasts. Presence of *P. oceanica* was also reported along the Dardanelles Strait and in the Marmara Sea.

Along the Moroccan coastline, *P. oceanica* was absent, except for the Chafarinas Islands (35°05′ N, 02°25′ E). According to limited information available, along the southern Mediterranean coast, the eastern boundary of *P. oceanica* meadows was located in Algeria, where they covered 4,072 ha, but the detailed distribution remains largely unknown. Furthermore, various maps and point data helped document the distribution of beds along the Tunisian coasts. *P. oceanica* was documented in the Gulfs of Gabès and Tunis, the Galite Archipelago, the Zembra Island and in other coastal areas, covering 518,685 ha. Along the Libyan coastline, meadows maps were available for lagoons (Farwà and Ain Al-Ghazala) and other scattered points, and the total covered area was 1,235 ha. The current *P. oceanica* distribution across the western Egyptian coasts was represented by point information only, whereas the meadows were surely absent off the Nile’s Delta and along the eastern coasts. The presence of meadows has never been confirmed along the coast of Syria and Lebanon, and has never been reported along the Israeli coastline. Therefore, *P. oceanica* could be considered absent along the coastlines of these eastern Mediterranean countries.

### Historical data and patterns of regression

Historical data on seagrass beds were available for Spain, France/Monaco, Italy, Albania, Tunisia, Egypt and Turkey. A detailed list of collated studies can be found in the Supplementary References and Supplementary Table S1 (see Supplementary Information online). The estimated lost area of *P. oceanica* was 124,091 ha over the past 50 years, which corresponds to an average regression of 10.1% of the total known area (Mediterranean basin). If we consider only those areas for which we had historical information (368,837 ha), the estimated loss of *P. oceanica* was 33.6%.

**Figure 5:**
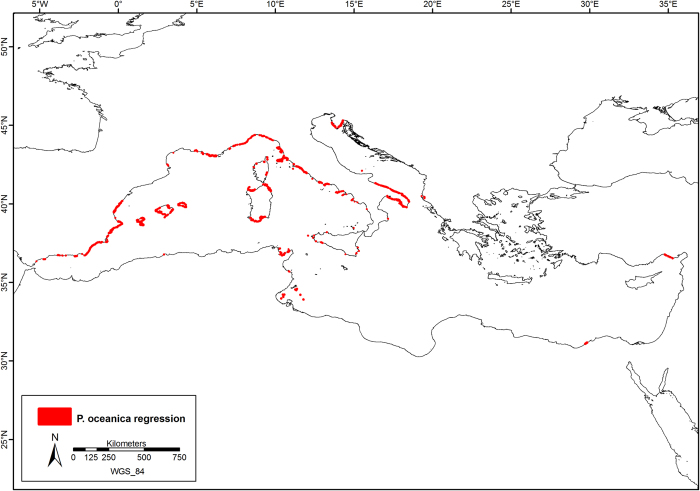
Coastline with regression of *Posidonia oceanica* meadows. Know areas with reported *P. oceanica* meadows loss (red areas) across the Mediterranean Sea over the last 50 years. Map created with ArcGIS^®^ software by Esri (Environmental Systems Resource Institute, ArcMap 9.3, www.esri.com) using data from OpenStreetMap.org (© OpenStreetMap contributors^59^).

Detailed information is reported in Table 2, in which the percentage of regression (total historical area compared to the total current area) of *P. oceanica* meadows and the time range of data are summarized for each country. The extent of regressive phenomena of *P. oceanica* meadows in the past 50 years, based on the comparison of historical and current maps available, is shown in Fig. 5.

**Figure 6:**
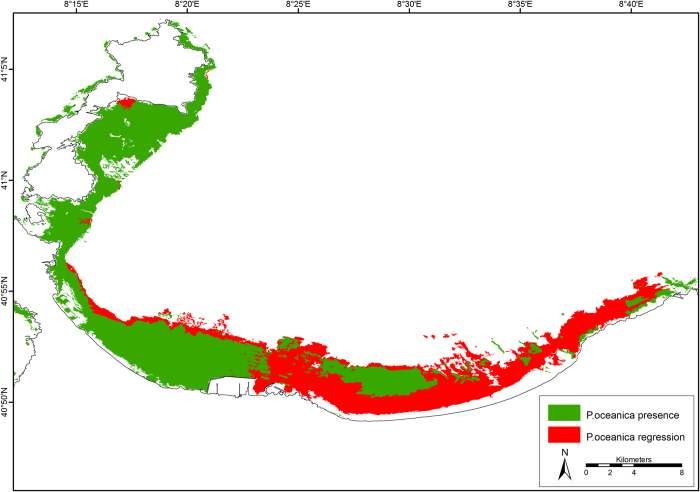
An example of the GIS output. *Posidonia oceanica* meadows in the Gulf of Asinara (Sardinia, Italy). The green areas represent the current distribution of *P. oceanica*, the red areas represent dead *matte*, which was used to estimate the regression. Map created with ArcGIS^®^ software by Esri (Environmental Systems Resource Institute, ArcMap 9.3, www.esri.com) using data from OpenStreetMap.org (© OpenStreetMap contributors^59^).

In France, current information on meadows was largely available and a standardized network (*Posidonia* Monitoring Network, PMN) has been monitoring and collecting data on its status^29^. In this country, historical maps were the oldest spatial data available for the Mediterranean Sea^16^ and other scientific publications testify meadow regressive phenomena caused by both natural and human impacts^11^. Despite uncertainties surrounding the precision of historical data, the collated maps allowed us to estimate a total loss of 2,753 ha of *P. oceanica* beds along the French continental coasts. In Corsica, regression was found to be very limited^21^. In Italy, broad meadow areas declined, and regression was documented along the continental coasts of Liguria, Tuscany, Latium and Apulia regions^20^,^19^, where a total regressed area of 34,472 ha was calculated over the last 20–30 years. In Sardinia, large areas of dead *matte*, amounting to 23,215 ha, were present in the Gulfs of Cagliari, Olbia and Asinara (see Fig. 6). Relevant signs of regression were documented in different areas of Spain^4^,^30^. According to historical data and maps available for the Spanish coastline, regressed *P. oceanica* meadows amounted to 49,585 ha over the last twenty years. Some current information was also available for the Eastern Mediterranean, showing regression in Vlora Gulf (Albania) where a loss of 907 ha was documented^31^. In the Maghreb region, historical information included a map of the Gulf of Tunis showing a regression of 13,159 ha^32^ and additional point data came from the Gulf of Gabès^33^. In Egypt, historical information was limited to the central part of the coastline, highlighting a fragmentation of meadows and their disappearance in El Agami harbour during the last decades. *P. oceanica* meadows seemed to have completely disappeared along the coast of Syria and Lebanon, but there the historical presence of *P. oceanica* has never been confirmed.

## Discussion

As part of this study, we collated all the spatial information available in order to create a baseline distribution map of *P. oceanica* meadows across the Mediterranean basin. Where possible, we showed the current areal extent of *P. oceanica* meadows and potential trajectories of change. In the past, several attempts to collate such data across the Mediterranean Sea took place, but the present study represents a joint Mediterranean-wide effort, resulting in a unified and as complete as possible vision of meadows distribution and trends of change. The EUSeaMap project^34^ produced a distribution map of marine habitats limited to the Western Mediterranean basin, including *P. oceanica* meadows, and used a broad geographical scale. The Barcelona Convention’s Regional Activity Centre for Specially Protected Areas (RAC/SPA) collated existing data and maps without creating a complete Mediterranean-scale map (see Supplementary References UNEP-MAP RAC/SPA 2009, Supplementary Information online). While Marbà *et al.*^4^ provided an evaluation of variations in the *P. oceanica* areal extent and cover, over the last 20 years, through only limited information on meadow changes. The MediSeH project^28^ can thus be considered the first large-scale effort (i) to collate all the knowledge available (current and historical) and (ii) to present a reliable and detailed distribution map of *P. oceanica* across the entire basin. This effort can represent the baseline for the future challenge of assessing “Good Environmental Status” of seagrasses and to fulfil European and International conservation targets for this regional sea.

Additionally, an effort was made to distinguish between areas where *P. oceanica* is absent and areas for which no data exist. This is a significant improvement since in the literature there are no maps about the absence of *P. oceanica* or the lack of data. Furthermore, all the cartography available on *P. oceanica* for the Mediterranean countries provided fragmented and approximate estimates of meadows distribution, or data at limited spatial resolution^25^. Such scattered or low resolution information is too unreliable for planning and setting up future management regimes for the coastal zone. However, there are remarkable differences concerning the quantity of spatial data available in different parts of the basin. In particular, the western part has much more information available than the eastern part, where the “absence” of data is common, though it does not necessarily mean that *P. oceanica* is absent.

Moreover, the MediSeH project can be considered the first effort to make an extensive collection of *P. oceanica* distribution data available online. Indeed, previous works^26^ provided online access to seagrass spatial data at a broad resolution. As part of the MediSeH project, the collated current and historical data on the *P. oceanica* distribution were stored within a geodatabase and were made available through the development of an online GIS data viewer (http://www.mareaproject.net/medviewer/), enabling their visualization.

Seagrass presence depends on a number of factors such as physical variables (e.g. temperature, salinity, depth, turbidity) which regulate its physiological activity, natural phenomena (climate change) and anthropogenic pressures. Meadows cover the majority of the Western Mediterranean coasts, but they are absent along parts of the Spanish coastline (Tarragona region), near the Ebro’s mouth, where freshwater input affects salinity and turbidity, thereby hindering seagrass growth^35^. Similarly, along the French coasts between the Albères coastline and the Rhone River’s mouth (except for small beds in Cape d’Adge), considerable contributions of freshwater, suspended sediment and organic matter input do not allow *P. oceanica* to develop into meadows^36^. *Posidonia oceanica* is also absent in Morocco (except for the Chafarinas Islands), probably due to the influence of cold Atlantic Water. In the Adriatic Sea, meadows are distributed along the eastern part (the coastline of Slovenia, Croatia, Montenegro and Albania)^37^, the Apulian coasts^38^ and small patches are present in Friuli (north-east of Italy)^39^. In the North and Central-Western Adriatic Sea (Italian coasts of Abruzzi, Marche, Emilia and Veneto), inputs of suspended sediment and dissolved organic matter from the Po’s outlet, create high levels of turbidity along the central-western Adriatic coast, all the way until the Gargano Promontory. In the Levant Sea (Syrian, Lebanese and Israeli coastline), early reports of *P. oceanica* should be considered erroneous because meadows are not present in this area.

The influence of temperature on *P. oceanica* growth was highlighted in several studies^40^ and the role of temperature in defining the eastern meadows boundaries was suggested by some authors^41^. So far, along the southern Turkish coasts, two reports only were available showing that *P. oceanica* was present in the bays of Iskenderum and Mersin^42^. In the same areas, recent publications showed that this species is no longer present and meadows end with a sharp border in the Levantine basin^43^. Along the southern Turkish coast, the average temperature in the eastern portion is warmer than the one in the western portion. Indeed, the ranges of water column temperature (up to 30 m depth), measured for the eastern part, were closer to the maximum temperature of the western side (respectively 27–29 °C and 23–28 °C)^41^. Considering these profiles, the separation point of temperature ranges for the western and eastern sides is located around 27.5 °C and this temperature seems to limit the growth of *P. oceanica* in the Levant Sea. Nevertheless, the maximum daily average temperature measured in the study area, where live meadows were found, was 28.4 °C^41^. This value was set as the Maximum Tolerable Temperature Limit and it was assumed to be the maximum temperature for *P. oceanica* growth in the Levant Basin^41^.

In Egypt, *P. oceanica* meadows are present along the western coast down to the Abu-Quir Bay. The absence of beds in the eastern part could be explained through a decrease of salinity and water transparency due to the considerable freshwater input from the Nile’s Delta.

An exception to the eastern boundary of meadows in the Levantine Sea was found in the waters surrounding Cyprus. In this area, *P. oceanica* creates meadows all around the island’s coasts^44^.

Surveys carried out in the Marmara Sea pointed out the presence of wide meadows along the Dardanelles Strait and isolated beds in the inner part of the basin^45^. Generally, *P. oceanica* is known to be a stenohaline species living in a salinity range between 36.5 and 39.5 ppt^46^. However, the salinity ranges near these beds are between 24 and 28 ppt in the Dardanelles Strait and between 21.5 and 26.5 ppt in the Marmara Sea. Based on oceanographic observations, this exceptional endurance to condition of low salinity shows that the currently isolated *P. oceanica* beds could be a relic population composed of genotypes adapted to brackish waters and growing colonially in isolated condition since the mid-Holocene^45^.

The comparison of current distribution maps with available historical ones allowed us to assess the changes undergone by meadows over time. As we mentioned in the Results, in areas for which historical data were available, the estimated regression of *P. oceanica* meadows was 34% in the last 50 years. With reference to the IUCN’s draft of Red List criteria for ecosystems^47^, that estimate makes *P. oceanica* habitat an “endangered” ecosystems.

The results of our work showed that the regression of meadows is a generalised phenomenon in the Mediterranean Sea, even though some exceptions exist (e.g. Corsica, parts of the Sardinian coastline and the Valencia region in Spain).

However, we should note that historical knowledge is generally fragmented, with different levels of accuracy across each Mediterranean country. The accuracy of historical maps can be questioned, both in terms of survey methods and restitution issues^14^, meaning that the percentage loss could be underestimated or overestimated. Our approach involved importing historical maps within a GIS system, scrutinizing the description of meadows’ limits and patterns, finding related scientific literature and observing the current maps, including considerations about the widespread presence of dead *matte*. This approach represents an effective way to combine information from different perspectives and quantify signs of an ongoing regression.

The differences between historical and current maps show an estimated reduction of 27.7% of the total seagrass area along the southern Latium coasts, in agreement with previously published figures^19^. In Spain, noteworthy signs of regressions were recorded in different regions, mainly due to human activities (e.g. illegal trawling, aquaculture farming)^48^. It has also been estimated that between 18% and 38% of potential meadows area may have been lost since 1960 s mostly in the Northwest Mediterranean basin^4^, in addition to marked declines along the Alicante region^30^. These assessments agree with our estimated regression of 29% for the continental and insular coasts, for which current and historical maps were available. However, a recent study which monitored *P. oceanica* meadows along the Valencia Region in Spain between 2002 and 2011 showed that the majority of meadows were either stationary or they have increased in density and covering^30^. This particular study suggests that the marked regression recorded in these sites has to be ascribed to the period from 1990 to 2000, whereas after that period the rate of regression has clearly slowed down up to the point of reversing in some areas. The research also highlighted the importance of long-time series in detecting possible changes in the population dynamics of this species.

A loss of 30% of the meadows was reported along the Ligurian coasts since the 1960 s^20^, whereas our estimate was 19%. In France, a *P. oceanica* regression of 23% was reported over the last 50 years, or, in more detail, 2% in Corsica (Cap Corse) in the last 15 years, 9.5% since the 1960’ (St. Florent), 4.3–5% in Marseille-Cortiou^49^ and 90% along the coast of Marseille in the last 100 years^11^. Our estimate for the French continental coast is equal to an average of 9%. In those cases where estimates differ, discrepancies are likely due to problems of accuracy of historical maps, which were identified by authors working in these areas^14^,^20^,^21^.

Looking at the regressive areas, the more severe situations occur in sites with a medium or high human impact (e.g. proximity to fishing ports, urbanised area, coast with altered sedimentary/hydrologic regimes), but also in proximity to river mouths which are located along the continental coastline (Central Tyrrhenian Sea, Spanish coasts). Along the coasts of offshore islands, the situation is generally stable (for example in Corsica), even though large regressive areas are evident along the coast of the main industrial and populated gulfs of Sardinia (Olbia, Cagliari, Asinara) and along coasts of the Balearic Islands.

The regressive trend of seagrasses is a phenomenon which has been observed along the majority of the world’s coasts^5^. The main causes are combinations of natural and human impacts (i.e. trawling, anchoring, fish farming, coastal constructions, warming, acidification, alien species invasion)^4^,^5^,^23^,^24^. A global review of scientific literature indicates that 29% of the known areal extent of seagrasses has disappeared since 1879^5^. In the Mediterranean Sea, meadows regression trends have already been reported since 1952 and early studies have suggested that it is due to a limited adaptation of the plant to the existing hydrological and climatic conditions in the Mediterranean Sea, mainly along the north-western coasts^50^. Later, the low genetic variability of this species was highlighted as a major contributor to the low resilience of *P. oceanica*; however, the availability of different powerful molecular markers revealed a higher meadow genetic variability than previously thought^51^,^52^. During the twentieth century and especially in the 1950 s, *P. oceanica* meadows have considerably regressed, mainly near large urban developments and ports such as Barcelona, Marseille, Toulon, Genoa, Trieste, Alexandria and Gabès^53^,^54^ Recently, it was suggested that the warming of the basin may lead to the functional extinction of *P. oceanica* meadows by 2050^55^.

The distribution map of *P. oceanica*, compiled by the MediSeH project and presented here, helps to fill the gap in knowledge on the presence and absence of *P. oceanica* meadows along the Mediterranean coasts. Furthermore, the work points out the lack of relevant data in different parts of the Mediterranean Sea, with particular reference to the eastern basin. Future mapping and monitoring efforts should target the remaining unmapped coastline (21,500 km) located in the southern and eastern regions of the basin, and in particular along the Algerian, Libyan, Croatian, Montenegrins, Greek and Turkish coasts. New surveys are expected to increase the known extent of *P. oceanica* meadows.

Despite the scarcity of information on the presence and distribution of *P. oceanica* meadows in some areas, a progressive regression seems to be taking place in those countries where the distribution is well known. However, this is a complex issue. The published literature shows a gradient of conditions ranging from marked regressive trends (up to 52% in some areas of Spain or 32% along the coastline of the Central Tyrrhenian Sea), to more localized regressive phenomena (the Ligurian Sea, Albania, Tunisia) and occasional stability or small increases of the meadows (Corsica and the Valencia region in Spain). Variability in trajectories of change among regions points out that regressive phenomena have to be mainly ascribed to cumulative effects of multiple local stressors^56^, rather than to processes at the Mediterranean basin scale, such as marine climate change^55^.

Moreover, the identified trend seems to be part of a large-scale phenomenon affecting seagrasses worldwide. The outcomes of our study strongly highlight the importance of implementing surveys specifically designed to assess the status both in the western and eastern Mediterranean countries, by means of continuous and coordinated monitoring over time, such as those already undertaken in some European countries (e.g. France). Despite the remaining data gaps, our effort of collating information on the distribution of *P. oceanica* and documenting patterns of regression shows that sufficient information exists to identify and prioritize areas where cost-effective schemes for threats reduction could be implemented. Now, the challenge is the identification of reversible threats that can be managed through specific actions capable of reversing present patterns of change and ensuring *P. oceanica* persistence at Mediterranean scale.

The broad distribution of *P. oceanica* in the Mediterranean Sea indicates that meadows are the result of ecological and evolutionary processes occurring over centuries. Time scales such as these are in contrast to the rapid and acute current impacts, caused directly or indirectly by human activity on seagrasses^57^. Indeed, the meadows are deteriorating at a higher rate than the one over which they spread during their development^58^, a trend that appears difficult to reverse, due to the low resilience of this slow-growing species.

## Methods

We looked for the literature showing current and past distribution maps (i.e. shapefiles, polygons), and point data of presence/absence of *P. oceanica* in different countries across the Mediterraneand basin. The Web of Science literature database was used, with search terms within the ‘Topic’ field such as “*Posidonia oceanica”* and “Distribution” or “Map” or “Regression” or “Decline” or “Progression” or “Recovery” or “Status” or “Cartography” or “Cover” or “Density”.

Governmental authorities (Ministries, Regional authorities) and other administrative offices of different countries were contacted to access information collated as part of Natura 2000, as well as local unpublished data (grey literature). We enriched the collated dataset using data obtained from national, EU and international projects’ websites. To fill the gaps in knowledge, local experts (e.g. researchers, civil servants in environmental ministries) were contacted for additional information. Ultimately, 263 studies were found to be relevant and these include reviewed papers, unpublished dataset, reports of EU or national projects and websites (see Supplementary Information and Supplementary Table S1 online).

Most of the spatial data were not available in geo-referenced digital format (e.g. shapefile, raster file) suitable for graphic software (e.g. ArcGIS, AutoCAD), but as paper maps only such as .jpeg and .pdf. The maps available had a great heterogeneity of spatial resolution, ranging from 1:1,000 to 1:250,000. Collated maps were characterised by various geographical projections, datum and legends, with a suite of non-standardised symbology. As a consequence, paper maps were manually digitized, the new maps geo-referenced (UTM Projection with WGS 1984 Datum) and incorporated in a GIS database (see Fig. 6 and Supplementary Figure S1 online).

Symbology was simplified and all symbols, which were used by different maps, were unified in two categories: (i) “*Posidonia oceanica*” and (ii) “dead *matte*”, regardless of the substrate (e.g. rock, sand or *matte*) or other meadow characteristics (e.g. mosaic of *P. oceanica* and rocks, *P. oceanica* and *Cymodocea nodosa*). However, the original information is still available within the GIS database. The remaining coastlines were classified as (iii) known absence of *P. oceanica* and (iv) “*no data areas*”, where no information on presence or absence was available.

All the collated data and maps were standardized using the Geographical Information System software ArcGIS^®^ software by Esri (Environmental Systems Resource Institute, ArcMap 9.3, http://www.esri.com). For the coastline and conspicuous points in the mainland, the OpenStreetMap contributors^59^ data coverage (http://www.openstreetmap.org/) was used. The bathymetric lines or points came from different local sources (i.e. Italian “Istituto Idrografico della Marina”), the GEBCO Project^60^, the EUSeaMap Project^34^, or, where present, from the original maps. Supplementary Figure S1 shows a detailed example of the current and historical distribution of *P. oceanica* and the bathymetric data available along the coast of the central Tyrrhenian Sea. We considered the most recent map of an area as the present *P. oceanica* distribution. Older maps available for the same area were considered to be historical information. Despite their low accuracy, these were included in the analysis only if they could be exactly geo-referenced. In fact, we estimated the level of accuracy of maps according to two criteria that are the maps’ format and the method of data geo-referencing^14^. Detailed maps provided in a geo-referenced digital format were considered as “accurate”. The reliability of paper maps was carefully estimated according to their initial scale and resolution^14^, and as a consequence, maps characterized by large-scales or low resolutions were excluded. Moreover, we considered as “accurate” maps acquired through modern navigation systems such as the GPS (Global Positioning System). In contrast, part of older historical cartography acquired by less accurate systems such as LORAN (LOng RAnge Navigation) were excluded, with the exception of distribution maps which reported detailed depth values or bathymetric lines that could be exactly geo-referenced (see Supplementary Figure S1 online).

After the standardization of maps and the creation of a shapefile representing the whole distribution of *P. oceanica* meadows, we estimated differences in seagrass extension by comparing current and historical maps we evaluated the extent of regression and calculated meadows’ variations caused by regressive phenomena. Where data and maps were available, regression values were calculated by ArcGIS^®^ software and listed in Table 2, reporting amounts of lost *P. oceanica* with relation to the coastline length for which current and historical information was available.

In some areas, the literature available on regressive status of *P. oceanica* was reviewed in order to gauge the reliability of our assessment or to improve the quality of the information of inaccurate maps. As a cross-check, data reported in the literature was compared to information found on maps, with special regard to the positioning of meadows’ lower limits. Remarkable discrepancies between historical and current maps were ignored if they were not confirmed by scientific papers or reports and therefore they were clearly caused by positioning or surveying errors. According to our assessment, we classified the Mediterranean coastline using the following categories: 1) areas with confirmed regression, 2) areas without regression and 3) areas without historical information. The first category also includes areas which have been classified as “dead *matte*” (see Fig. 6), despite the absence of historical data for these localities. In fact, we considered the visible dead *matte* as a result of natural or anthropogenic effects that have occurred in the last decades. Moreover, we assumed that dead *matte*, resulting from comparatively older events (e.g. sea level variations), should have disintegrated and thus would not be visible anymore.

At a later stage, all the collated and standardized data were incorporated in a GIS database and they were made accessible on the MediSeH online GIS data viewer (http://www.mareaproject.net/medviewer/).

The GIS database development was performed in a two-steps procedure. First, an empty geodatabase was created using an ArcGIS^®^ software and all the information was incorporated. Second, the geodatabase was transferred to a geoserver for online visualisation through a Web Map Service (WMS). The MediSeH online GIS data viewer was developed using ALOV Map^®^ (http://prof.if.ktu.lt:8090/alovmap/), a multi-platform, portable Java^®^ application for online publication of cartographic datasets and interactive on last generation web-browser. The final online GIS data viewer was highly customised in order to improve and facilitate the visualization and scalable selection of the extensive number of assembled shapefiles and georeferenced images from the produced datasets. The created MediSeH webGIS represents a user-friendly for the visualization of all the gathered data on the current *P. oceanica* distribution, the magnitude of regressive phenomena and the areas with lack of information throughout the Mediterranean Sea.

## Additional Information

**How to cite this article**: Telesca, L. *et al.* Seagrass meadows (*Posidonia oceanica*) distribution and trajectories of change. *Sci. Rep.* **5**, 12505; doi: 10.1038/srep12505 (2015).

## Supplementary Material

Supplementary Information

Supplementary Information

## Figures and Tables

**Table 1:** Spatial extent of *Posidonia oceanica* meadows across the Mediterranean Sea.

	**Mediterranean Sea**	**Western basin**		**Eastern basin**	
Coastline length (km)	46,000	11,621	25%	34,379	75%
Coastline length with *P. oceanica* (km)	11,907	6,201	14%	5,706	12%
Coastline length without *P. oceanica* (km)	12,622	3,925	9%	8,697	19%
Coastline length without data (km)	21,471	1,494	3%	19,977	43%
Total area of *P. oceanica* (ha)	1,224,707	510,715	41.7%	713,992	58.3%

**Table 2:** Lengths of coastline with the known current and historical presence of *Posidonia oceanica*, the percentage of regression and the time range of data.

**Country**	**Currently surveyed coastline (%)**	**Historical surveyed coastline (%)**	***P. oceanica* total current area (ha)**	***P. oceanica*total historical area (ha)**	***P. oceanica*regression (%)**	**Time range of data**
Spain	100%	70%	172,669	222,254	29%^1^	1993–2011
France/Monaco	100%	60%	94,030	96,783	9%^2^	1980–2011
Italy	100%	42%	337,611	395,298	25%^3^	1990–2005
Slovenia	100%	—	9	—	—	2004
Croatia	14%^4^	—	31,437	—	—	2010
Montenegro	100%^5^	—	—	—	—	2004
Albania	100%	—	4,803	5,710	16%	2007–2008
Malta	100%	—	5,860	—	—	2002
Greece	8%^4^	—	44,939	—	—	2011
Turkey	29%^4^	6%	287	—	—	2009
Cyprus	30%^4^	—	9,040	—	—	2008
Syria, Lebanon, Israel	100%	Absent^6^	Absent^6^	—	—	2003
Egypt	63%^5^	3%	—	—	—	2006
Libya	11%^4^	—	1,235	—	—	2011
Tunisia	81%^4^	13%	518,685	531,844	2%	1972–2010
Algeria	16%^4^	—	4,072	—	—	2010
Morocco	100%^5^	—	—	—	—	2006

^1^Andalucia, Murcia, Valenciana regions, the Balearic Islands

^2^the continental coasts of France/Monaco

^3^Liguria, Tuscany, Latium, Apulia and Sardinia regions

^4^Polygons and point data: surface area referred to polygons only

^5^Point data only: no surface area is available

^6^Confirmed *P. oceanica* absence.
