# Supplementary material for: Seagrass meadows (Posidonia oceanica) distribution and trajectories of change
Source: Sci Rep. 2015 Jul 28;5:12505. doi: 10.1038/srep12505 (PMC4516961; doi:10.1038/srep12505)
Supplement: Supplementary Information [file srep12505-s1.pdf]

SUPPLEMENTARY INFORMATION

Seagrass meadows (*Posidonia oceanica*) distribution and trajectories of change

Luca Telesca, Andrea Belluscio, Alessandro Criscoli, Giandomenico Ardizzone, Eugenia T. Apostolaki, Simonetta Fraschetti, Michele Gristina, Leyla Knittweis, Corinne S. Martin, Gérard Pergent, Adriana Alagna, Fabio Badalamenti, Germana Garofalo, Vasilis Gerakaris, Marie Louise Pace, Christine Pergent-Martini & Maria Salomidi.

1. **Supplementary Figure S1.** An example of GIS output

2. **Supplementary References.** Sources of data on the distribution of *Posidonia oceanica*

3. **Supplementary Table S1.** (Excel spreadsheet). Subset of the Supplementary References that are linked to a specific geographic area (specific Country or Mediterranean region)

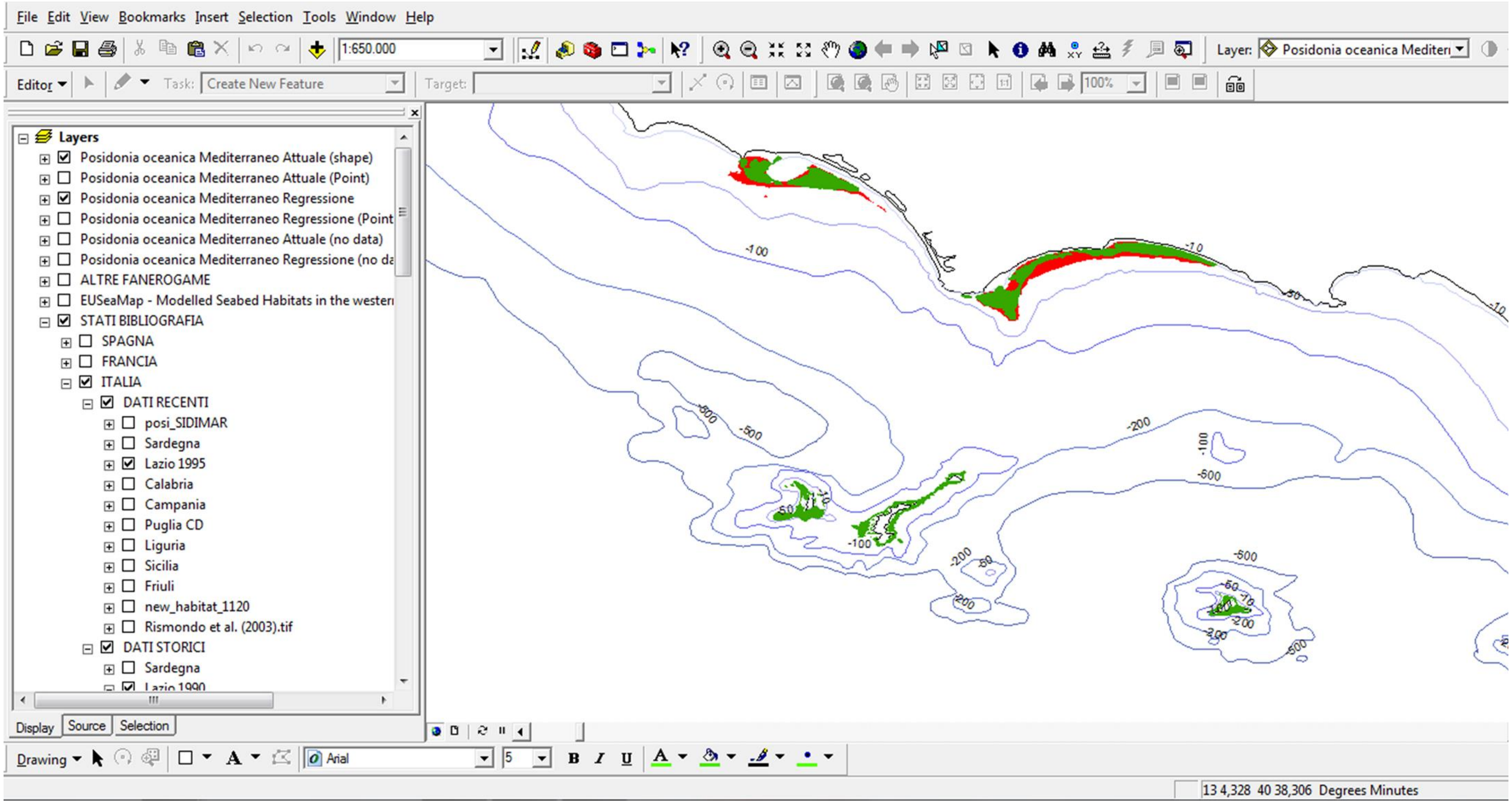

Supplementary Figure S1. An example of the GIS output. *Posidonia oceanica* meadows along the coast of Latium (Italy, Central Tyrrhenian Sea): in green the current distribution of *P. oceanica*[19], in red the negative difference (lost *P. oceanica*) based on a historical map (see Ardizzone, G.D. & Belluscio, A. (1996), Supplementary References) that was used to estimate the regression. Bathymetries, blue lines, from the "Instituto Idrografico della Marina, Italy". Map created with ArcGIS® software by Esri (Environmental Systems Resource Institute, ArcMap 9.3, www.esri.com) using data from OpenStreetMap.org (© OpenStreetMap contributors[59]).

Supplementary References. Sources of data on the distribution of *Posidonia oceanica*

<u>Scientific and Grey Literature</u>

AA-VV. in *Inventario e cartografia delle praterie di Posidonia nei compartimenti marittimi di Manfredonia, Molfetta, Bari, Brindisi, Gallipoli e Taranto: relazione analitica finale* (eds ASSOPESCA & COISPA Tecnologia e Ricerca) 204 pp. (Regione Puglia, 2006).

ANDROMEDE. in *Etude et cartographie des biocénoses marines de l'archipel de la Galite, Tunisie. Initiative pour les petites îles de Méditerranée* 132 pp. (Contrat OEil d'Andromède-Agence de l'eau, 2010).

ANDROMEDE. in *Etude et cartographie des biocénoses marines de l'île de Zembra, Tunisie. Initiative pour les petites îles de Méditerranée* 122 pp. (Contrat OEil d'Andromède-Agence de l'eau, 2010).

Antonic, O. *et al.* Mapping the habitats of the Republic of Croatia (2000- 2004): the project overview (in Croatian). *Drypis: J. Appl. Ecol.* **1**, 1-40 (2005).

Ardizzone, G. & Migliuolo, A. Modificazioni di una prateria di *Posidonia oceanica* (L.) Delile del medio Tirreno sottoposta ad atività di pesca a strascico. *Naturalista Sicil.* **4,** 509-515 (1982).

Ardizzone, G. D. & Belluscio, A. in *Le praterie di Posidonia oceanica lungo le coste laziali in Il Mare Del Lazio* 194-217 (University of Rome "La Sapienza", 1996).

Ardizzone, G. D. & Pelusi, P. Regression of a tyrrhenian *Posidonia oceanica* prairie exposed to nearshore trawling. *Rapp. Comm. int. Mer Médit.* **28**, 175-177 (1983).

Ardizzone, G., Belluscio, A. & Maiorano, L. Long-term change in the structure of a *Posidonia oceanica* landscape and its reference for a monitoring plan. *Mar. Ecol.* **27,** 299-309 (2006).

ARPAM, *Rapporto sullo stato di qualità ambientale della fascia costiera marchigiana: balneabilità e biocenosi. Rapporto conclusivo.* (2003) Available at: http://www.autoritabacino.marche.it/. (Accessed: 5th March 2015)

Astier, J. M. Cartographie des fonds marins de la région de Toulon par le groupe ECOMAIR. *Ann. Soc. Sci. nat. Archéol.* **27,** 1-15 (1975).

Astier, J. M. Impacts des aménagements littoraux de la rade de Toulon, liés au techniques d'endigage, sur les herbiers à *Posidonia oceanica* in *International Workshop on Posidonia Oceanica Beds* (eds Boudouresque, C. F., Jeudy de Grissac, A. & Olivier, J.) 255-259 (GIS-Posidonie, 1984).

Astier, J. M. Régression de l'herbier de *Posidonies* en rade des Vignettes à Toulon. *Ann. Soc. Sci. nat. Archéol.* **24**, 97-103 (1972).

Augier, H. & Boudouresque, C. F. Dix ans de recherches dans la marine du Parc National de Port-Cros (France). Toisième partie. *Ann. Soc. Sci. Nat. Archéol.* **27,** 133-170 (1975).

Augier, H. & Boudouresque, C. F. Végétaton marine de l'ile de Port-Cros. V. La baie de port-man et le problème de la régression de l'herbier de *Posidonies*. *Bull. Mus. Hist. Na. Marseille* **30,** 145-164 (1970).

AULA DEL MAR-MALAGA, *Praderas de Fanerógamas marinas en el litoral andaluz*. (1998) Available at: http://www.juntadeandalucia.es/medioambiente/. (Accessed: 5th March 2015)

Badalamenti, F. *et al.* Effects of dredging activities on population dynamics of *Posidonia oceanica* (L.) Delile in the Mediterranean sea: the case study of Capo Feto (SW Sicily, Italy). *Hydrobiologia* **555,** 253-261 (2006).

Badalamenti, F., Alagna, A., D'Anna, G., Terlizzi, A. & Di Carlo, G. The impact of dredge-fill on Posidonia oceanica seagrass meadows: regression and patterns of recovery. *Mar. Pollut. Bull.* **62,** 483-489 (2011).

Bakran-Petricioli, T. & Schultz, S. T. Biometry and leaf lepidochronology of the seagrass *Posidonia oceanica* in the Croatian Adriatic in *Proceedings of 4th Mediterranean Symposium On Marine Vegetation* (eds El Asmi, S. *et al.*) 28-31 (UNEP-MAP-RAC/SPA, 2010).

Bakran-Petricioli, T., Antoni, O., Bukovec, D., Petricioli. D. & Janeković, I. Modelling spatial distribution of the Croatian marine benthic habitats. *Ecol. Model.* **191**, 96–105 (2006)

Bakran-Petricioli, T., Schultz, S., Petricioli, D. & Kruschel C. Applicability of mediterranean baselines of *Posidonia oceanica* descriptors for the assessment of its conservation status along the eastern part of the Adriatic Sea in *Proceedings of 5th Mediterranean Symposium On Marine Vegetation* (eds Langar, H., Bouafif, C. & Ouerghi, A.) 198-199 (UNEP-MAP-RAC/SPA, 2014).

Balduzzi, A. *et al.* Valutazione dello stato di alcune praterie di *Posidonia* del mare Ligure sottoposte a diverse condizioni di inquinamento in *International Workshop on Posidonia oceanica Beds* (eds Boudouresque, C.F., de Grissac, A.J. & Oliver, J.) 63 – 72 (GIS Posidonie, 1984).

Ballesta, L. *et al.* Distribution and dynamics of *Posidonia oceanica* beds along the Albères coastline. *Ecology* **323**, 407-414 (2000).

Ballesteros, E., Cebrian, E. & Alcoverro, T. Mortality of shoots of *Posidionia oceanica* following meadow invasion by the red alga Lophociadia lallemandii. *Bot. Mar.* **50,** 8-13 (2007).

Bavestrello, G. Nuove osservazioni sulle praterie di *Posidonia oceanica* (L.) Delile del versante occidentale del Golfo Marconi (Riviera ligure di Levante). *Boll. Mus. Ist. Biol. Univ. Genova* **50-51,** 99-104 (1982).

Belsher, T., Houlgatte, E. & Boudouresque, C. F. Cartographie de la prairie à *Posidonia oceanica* et des principaux faciès sédimentaires marins du Parc national de Port-Cros (Var, France, Méditerranée). *Sci. Rep. of Port-Cros Nation. Park* **21**, 19-28 (2005).

Ben Alaya, H. Repartition et condition d'installation de *Posidonia oceanica* Delile et *Cymodocea nodosa* Ascherson dans le Golfe de Tunis. *Bull. Instit. Nation. Peche de Salammbo* **2,** 331-416 (1972).

Benedito, V. *et al.* Distribution and preliminary evaluaion of the state of the *Posidonia oceanica* on the Coasts of the Gulf of Valencia (Spain, Western Mediterranean). *Rapp. Comm. int. Mer Médit.* **32** (1990).

Bianchi, C. N. & Peirano, A. in *Atlante delle fanerogame marine della Liguria. Posidonia oceanica e Cymodocea nodosa* 146 pp. (ENEA-Centro Ricerche Ambiente Marino, 1995).

Borg, J. A. & Schembri, P. J. The state of *Posidonia oceanica* (L.) Delile meadows in the Maltese Islands (Central Mediterranean). *Rapp. Comm. Int. Mer Medit.* **34**, 123 (1995).

Borg, J. A., Rowden, A. A., Attrill, M. J., Schembri, P. J. & Jones, M.B. Occurrence and distribution of different bed types of seagrass *Posidonia oceanica* around the Maltese Islands. *Mediterr. Mar. Sci.* **10,** 45-61 (2009).

Boudouresque, C. F. & Meinesz, A. Decouverte de l'herbier de *Posidonie*. *Cah. Parc. nation. Port-Cros* **4**, 1-79 (1982).

Boudouresque, C. F. *et al.* A monitoring network based on the seagrass *Posidonia oceanica* in the North Western Mediterranean Sea. *Biol. Mar. Mediterr.* **7,** 328-331 (2000).

Boudouresque, C. F. *et al.* Monitoring methods for *Posidonia oceanica* meadows in Provence and French Riviera. *Sci. Rep. Port-Cros Nation. Park* **22,** 17–38 (2007).

Boudouresque, C. F. *et al.* in *Protection and conservation of Posidonia oceanica meadows* 202pp. (RAMOGE & RAC/SPA, 2012).

Boudouresque, C. F. in *Impact de l'homme et conservation du milieu marin en Méditerranée 2nd edn* (GIS Posidonie, 1996).

Boudouresque, C. F., Bernard, G., Pergent, G., Shili, A. & Verlaque, M. Regression of Mediterranean seagrasses caused by natural processes and anthropogenic disturbances and stress: a critical review. *Bot. Mar.* **52,** 395–418 (2009).

Boudouresque, C. F., Meinesz, A. & Lefevre, J. R. Cartographie des peuplements benthiques marins de Corse: I. La formation récifale à *Posidonia oceanica* de Saint Florent. *Ann. I. Oceanogr. Paris.* **61**, 27-38 (1985).

Bourcier, M. in *Regression des herbiers à Posidonia oceanica (L.) Delile, à L'est de Marseille, sous l'action conjuguée des activités humaines et des modifications climatiques, International Workshop on Posidonia oceanica beds* (GIS-Posidonie, 1989).

Buia, M. C., Silvestre, F., Iacono, G. & Tiberti, L. in *Identificazione delle biocenosi di maggior pregio ambientale al fine della classificazione della qualità delle acque costiere. Metodologie per il rilevamento e la classificazione dello stato di qualità ecologico e chimico delle acque, con particolare riferimento all'applicazione del decreto legislativo 152/99* (APAT, 2005).

Buskovic, V., Macic, V., Saveljic, D. & Ivanovic, A. in *Montenegro National Action Plan 1: Inventory and mapping of sensitive areas in National Action Plan (SAP BIO), UNEP-RAC/SPA and the Republic of Montenegro* 42 pp. (Ministry of Environmental Protection and Physical Planning, 2004).

Buskovic, V., Macic, V., Saveljic, D. & Ivanovic, A. in *National action plans produced within the project strategic action programme for the conservation of ciological diversity in the Mediterranean Region (SAP BIO)* 42 pp. (UNEP-MAP, 2004).

Calleja, M. L., Marbà, N. & Duarte, C. M. The relationship between seagrass (*Posidonia oceanica*) decline and sulfide porewater concentration in carbonate sediments. *Est. Coast. Shelf* **73**, 583–588 (2007).

Cameron, A. & Askew, N. in *EUSeaMap - Preparatory Action for development and assessment of a European broad-scale seabed habitat map final report. EC contract no. MARE/2008/07.* 240 pp. (JNCC, 2011).

Cancemi, G. *et al.* Indagine cartografica sulla prateria a *Posidonia oceanica* (L.) delile di Capo S. Marco (golfo di Oristano), mediante elaborazione di immagini fotoaeree. *Biol. Mar. Medit.* **4,** 472-474 (1997).

Cancemi, G., De Falco, G. & Pergent, G. Effects of organic matter input from a fish farming facility on a *Posidonia oceanica* meadow. *Estuar. Coast. Shelf S.* **56,** 961-968 (2003).

Cinelli, F. & Piazzi, L. in *Mappatura delle praterie di Posidonia oceanica (L.) Delile lungo le coste toscane* 80 pp. (CIBM, 1990).

Cinelli, F., Pardi, G., Papi, I. & Benedetti-Cecchi, L. Mappatura delle praterie a *Posidonia oceanica* (L.) Delile intorno alle isole minori dell'Arcipelago Toscano. *Att Soc. Tosc. Sci. Nat.* **102**, 93-110 (1995).

Coll, J. *et al.* in *Evaluación y control ambiental de algunos indicadores del estado de conservación del medio bentónico en aguas del Parque Nacional Marítimo-Terrestre del Archipiélago de Cabrera* 31pp. (Pandion, Consultoria ambiental, 1999).

Costantino, G. *et al.* Distribution and bio-ecological features of *Posidonia oceanica* meadows along the coasts of the southern Adriatic and northern Ionian Seas. *Chem. Ecol.* **26**, 91-104 (2010).

Darmoul, B. Pollution dans le Golfe de Gabes (Tunisie): bilan des six anné es de surveillance (1976-1981). *Bull. Inst. Natl. Sci. Tech. Oceanogr. Peche Salammbo* **15**, 61-84 (1988).

Darmoul, B., Hadj Ali Salem, M. & Vitello, P. Effets des rejets industriels de la région de Gabès (Tunisie) sur le mileu marin récepteur. *Bull. Inst. natn. tech. Oceanogr. Pêche Salammbo* **7,** 5-61 (1980).

de Falco, G. *et al.* Photo-aerial image processing and sediment analysis as indicators of environmental impact on *Posidonia oceanica* in the Mediterranean sea. *Biol. Mar. Medit.* **7,** 349-352 (2000).

de Villèle, X. & Verlaque, M. Changes and degradation in a *Posidonia oceanica* bed invaded by the introduced tropical alga Caulerpa taxifolia in the North Western Mediterranean. *Bot. Mar.* **38,** 79-87 (1995).

Delgado, O. *et al.* Effects of fish farming on seagrass (*Posidonia oceanica*) in a Mediterranean bay: seagrass decline after organic loading cessation. *Oceanol. Acta* **22,** 109-117 (1999).

Delgado, O. *et al.* Seagrass regression caused by fish cultures in Fornells Bay (Menorca, Western Mediterranean). *Oceanol. Acta* **20**, 557-563 (1997).

Delgado, O., Ruiz, J., Perez, M., Romero, J. & Ballesteros, E. Effects of fish farming on seagrass (*Posidonia oceanica*) in a Mediterranean bay: seagrass decline after organic loading cessation. *Oceanol. Acta* **2**, 109-117 (1999)

Di Carlo, G., Badalamenti, F., Jensen, A., Koch, E. & Riggio, S. Colonisation process of vegetative fragments of *Posidonia oceanica* (L.) Delile on rubble mounds. *Mar. Biol.* **147**, 1261–1270 (2005).

Diaz-Almela, D. *et al.* Patch dynamics of the Mediterranean seagrass *Posidonia oceanica*: implications for recolonisation process. *Aquat. Bot.* **89**, 397–403 (2008).

Díaz-Almela, E. *et al.* Benthic input rates predict seagrass (*Posidonia oceanica*) fish farm-induced decline. *Mar. Pollut. Bull.* **56**, 1332–1342 (2008).

Díaz-Almela, E., Marbà, N., Martínez, R., Santiago, R. & Duarte, C. M. Seasonal dynamics of *Posidonia oceanica* in Magalluf Bay (Mallorca, Spain): temperature effects on seagrass mortality. *Limnol. Oceanogr.* **54**, 2170–2182 (2009).

Diviacco, G., Spada, E. & Virno Lamberti, C. in *Le fanerogame marine del Lazio. Descrizione e cartografia delle praterie di Posidonia oceanica e dei prati di Cymodocea nodosa* 113 pp. (ICRAM, 2001).

Duarte, C. M. The future of seagass meadow. *Environ. Conserv.* **29**, 192-196 (2002).

El Lakhrach, H., Hattour, A., Jarbouio, O., Elhasni, K. & Ramos-Espala, A. A. Spatial distribution and abundance of the megabenthic fauna community in Gabes Gulf (Tunisia, eastern Mediterranean Sea). *Mediterr. Mar. Sci.* **13,** 12-29 (2012).

ESGEMAR. *Vegetación Submarina. Fanerógamas marinas y algas de interés general del litoral andaluz. Año 2003. Escala 1:50.000. Consejería de Medio Ambiente, Junta de Andalucía.* (2003) Available: www.juntadeandalucia.es/medioambiente/rediam/descargas_informacion_ambiental. (Accessed: 5th March 2015)

Falconetti, C. & Meinesz, A. Charting the seaward limit of *Posidonia oceanica* meadows and of circalittoral biocoenoses along the coast of Monaco. *Oceanol. Acta* **12**, 443-447 (1989).

Fernández Torquemada, Y., González Correa, J. M., Martínez J. E. & Sánchez Lizaso J. L. Evaluation of the effects produced by the construction and expansion of marinas on *Posidonia oceanica* (L.) Delile meadows. *J. Coast. Res.* **94,** 94 – 99 (2005).

Fernandez-Torquemada, Y. et al. Descriptors from *Posidonia oceanica* (L.) Delile meadows in coastal waters of Valencia, Spain, in the context of the EU Water Framework Directive. *J. Mar. Sci.* **65**, 1492–1497 (2008).

Foulquié, M. & Dupuy de la Grandrive, R. *Site Natura 2000 FR 910 1414 "Posidonies du Cap d'Agde". Inventaire de l'existant et analyse ecologique.* (2003) Abailable at: http://www.languedoc-roussillon.developpement-durable.gouv.fr/. (Accessed: 5th March 2015)

García-Chartón, J. A. *et al.* Respuesta de la pradera de *Posidonia oceanica* y su ictiofauna asociada al anclaje de embarcaciones en el Parque Nacional de Port-Cros (Francia). Publicaciones Especiales. *Instituto Español de Oceanografía* **11,** 423-430 (1994).

GAS/MEPA. *Baseline survey of the extent and character of Posidonia oceanica (L.) Delile meadows in the territorial waters of the Maltese islands, Final report* (2003). Available at: https://www.mepa.org.mt/SOER_Documents/Posidonia_survey.pdf. (Accessed: 5th March 2015)

Giakoumi, S. *et al.* Ecoregion-based conservation planning in the Mediterranean: dealing with large- scale heterogeneity. *PLoS One* **8,** e76449 (2013).

Gobert, S. *et al.* Assessment of the ecological status of Mediterranean French coastal waters as required by the Water Framework Directive using the *Posidonia oceanica* Rapid Easy Index: PREI. *Mar. Pollut. Bull.* **58,** 1727–1733 (2009).

Gonzalez-Correa, J. M. *et al.* Recovery of deep *Posidonia oceanica* meadows degraded by trawling. *J. Exp. Mar. Biol. Ecol.* **320**, 65–76 (2005).

Gonzalez-Correa, J. M., Fernández-Torquemada, Y. & Sánchez-Lizaso, J. L. Long-term effect of beach replenishment on natural recovery of shallow *Posidonia oceanica* meadows. Estuarine coastal and shelf science **76,** 834-844 (2008).

Gonzalez-Correa, J. M., Sempere, J. T. B., Sánchez-Jerez, P. & Valle, C. *Posidonia oceanica* meadows are not declining globally. Analysis of population dynamics in marine protected areas of the Mediterranean Sea. *Mar. Ecol-Prog. Ser.* **336,** 111-119 (2007).

Green, E. P. & Short, F. T. *World Atlas of Seagrasses* 310 pp. (University of California Press, 2003).

Gucu, G. & Gucu, A. C. Ecological significance of sea grass meadows (*Posidonia oceanica* (L.) Delile) in Bozyazı-Kızılliman marine protected area in *Oceanography of the Eastern Mediterranean and Black Sea* (ed Yilmaz, A.) 924-930 (Tubitak, 2003).

Guidetti, P. Population dynamics of *Posidonia oceanica* in the Ligurian Sea (Italy, NW Mediterranean): Evaluating the meadow health status by reconstructing methods. *Thalassia Salentina*, **27**, 33-46 (2004).

Guillén Nieto, J. E. *et al.* Antitrawling reefs and the protection of *Posidonia oceanica* (L.) delile meadows in the western Mediterranean Sea: demands and aims. *B. Mar. Sci.* **552,** 645-650 (1994).

Haritonidis, S. & Diapoulis, A. Evolution of Greek marine phanerogam meadows over the last 20 years. *Posidonia newsletter* **3**, 5-10 (1990).

Haritonidis, S., Diapoulis, A. & Nikolaidis G. First results on the localistation of the herbiers of marine phanerogams in the Gulf of Termaikos. *Posidonia Newsletter* **3,** 11-18 (1990).

Holmer, M., Marbà N., Lamonte, M. &Duarte C. M. Deterioration of sediment quality in seagrass meadows (*Posidonia oceanica*) invaded by macroalgae (*Caulerpa* sp.). *Estuar. Coast.* **32,** 456-466 (2009).

Instituto de Ecología Litoral. in *Posidonia oceanica. Redes de seguimiento y estado de conservación en el Mediterráneo español* 128 pp. (Diputación de Alicante, 2009).

Jakl, Z. Large scale marine habitat and species mapping on the Croatian side of the Adriatic Sea. *Varstvo Narave* **1**, 79-98 (2011).

Jorda, G., Marbà, N. & Duarte C. M. Mediterranean seagrass vulnerable to regional climate warming. *Nat. Clim. Change* **2,** 821–824 (2012).

Kosontini, M., Djellouli, A. S., Langar, H. & Abdeljaoued, S. L'herbier a *Posidonia oceanica* de Port-Princes (CapBon, Tunisie): etude et presentation d'un patrimoine naturel in *Proceedings of the third Mediterranean symposium on marine vegetation, France, Marseille, 27-29 March 2007* (eds Pergent-Martini, C., El Asmi, S. & Le Ravallec, C.) 274-276 (RAC/SPA, 2007).

Leriche, A. *et al.* Spatial, temporal and structural variations of a *Posidonia oceanica* seagrass meadow facing human activities. *Aquat. Bot.* **84**, 287-293 (2006).

Leriche, A., Boudouresque, C.F., Bernard, G., Bonhomme, P. & Denis, J. A one-century suite of seagrass bed maps: can we trust ancient maps? Estuar. Coast. Shelf S. 59, 353-362 (2004).

Lopez y Royo, C. in *Utilisation de Posidonia oceanica (L.) Delile comme outil de gestion de la qualité écologique du milieu marin. PhD Thesis.* 242 pp. (Université de Corse, 2008)

Lopez y Royo, C., Pergent, G., Pergent-Martini, C. & Casazza, G. Seagrass (*Posidonia oceanica*) monitoring in western Mediterranean: implications for management and conservation. *Environ. Monit. Assess* **171**, 365–380 (2010).

Lopez y Royo, C., Silvestri, C., Salivas-Decaux, M., Pergent, G. & Casazza, G. Application of an Angiosperm-based classification system (BiPo) to Mediterranean coastal waters: using spatial analysis and data on metal contamination of plants in identifying sources of pressure. *Hydrobiologia* **633**, 169-179 (2009).

Loquès, F. La limite inférieure de l'herbier de *Posidonies* des iles de Lérins (Baie de Cannes, France) traitement cartographique sur S.I.G. (systemes d'informations geographiques). *Mésogée* **54**, 22-34 (1995).

Luque del Villar, A. A. Inventario, evaluación y programa de mantenimiento- conservación de hábitats de interés prioritario del litoral de Almería: praderas de *Posidonia oceanica* in *Los tipos de Hábitat de Interés Comunitario en España, Guía Básica* (eds Bartolomé, C., Álvarez, J. & Vaquero J.) (Ministerio de Medio Ambiente, Dirección General para la Biodiversidad, 1996).

Maggi, P. Le problème de la disparition des herbiers de *Posidonies* dans le Golfe de Giens. *Sci. Pêche, Bull. Inst. Pêches Marit.* **221**, 7-20 (1973).

Maggi, P. Les herbiers à *Posidonies* et la pollution urbaine dans le golfe de Giens (VAR). *Ann. Inst. Michel Pacha* **5**, 1-11 (1972).

Maiorano, P. *et al.* Bioecological study of the benthic communities on the soft bottom of the Vlora Gulf (Albania). *J. Coastal Res.* **58**, 95–105 (2011).

Mancusi, C., Cecchi, E., Galloni, F., Padovani, S. & Serena, F. La mappatura di *Posidonia oceanica* in due aree della Toscana. *Biol. Mar. Mediterr.* **18**, 314-315 (2011).

Mansour, H. & Mostafa, H. M. Extention of *Posidonia oceanica* meadows in the Mediterranean waters of Egypt: historical review in *Proceedings of the first Mediterranean symposium on marine vegetation*: *Ajaccio, 3-4 October 2000* (eds UNEP-MAP-RAC/SPA) 163-167 (RAC/SPA, 2000).

Marbà, N. & Duarte, C. M. Interannual changes in seagrass (*Posidonia oceanica*) growth and environmental change in the Spanish Mediterranean littoral zone. *Limnol. Oceanogr.* **42,** 800-810 (1997).

Marbà, N. *et al.* Assessing the effectiveness of protection on *Posidonia oceanica* populations in the Cabrera National Park (Spain). *Environ. Conserv.* **29,** 509–518 (2002).

Marbà, N. *et al.* Direct evidence of imbalanced seagrass (*Posidonia oceanica*) shoot population dynamics in the Spanish Mediterranean. *Estuaries* **28,** 53-62 (2005).

Marba, N. *et al.* Effectiveness of protection of seagrass (Posidonia oceanica) populations in Cabrera National Park (Spain). *Environ. Conserv.* **29,** 509–518 (2002).

Marba, N. *et al.* Growth and population dynamics of *Posidonia oceanica* on the Spanish Mediterranean coast: elucidating seagrass decline. *Mar. Ecol-Prog. Ser.* **137**, 203–213 (1996).

Marbà, N. & Duarte, C. M. Mediterranean Warming Triggers Seagrass (*Posidonia oceanica*) Shoot Mortality. *Glob. Change Biol.* **16,** 2366–2375 (2010).

Marbà, N., Diaz-Almela, E. & Duarte C. M. Mediterranean seagrass (*Posidonia oceanica*) loss between 1842 and 2009. *Biol. Conserv.* **176,** 183-190 (2014).

Marbà, N., Díaz-Almela, E. & Duarte, C. M. *Posidonia oceanica changes in the Mediterranean Sea* (2013). Available at: http://digital.csic.es/handle/10261/88165. (Accessed: 5th March 2015)

Martín, M. A. *et al.* Cuantificación del impacto de las artes de arrastre sobre la pradera de *Posidonia oceanica* (L.) Delile, 1813. *Publicaciones especiales Instituto Español de Oceanografía* (1997).

Mas, J., Franco, I. & Barcala, E. Primera aproximación a la cartografía de las praderas de *Posidonia oceanica* en las costas mediterráneas españolas. Factores de alteración y de regresión. Legislación. *Publ. espec. Inst. Esp. Oceanogr*. **11**, 111-122 (1993).

MATT. in *Mappatura delle praterie di Posidonia oceanica e di altre fanerogame marine lungo le coste della Campania e della Calabria e delle isole minori circostanti. Relazione finale,* Vol. 1, 531 pp. (Roma: Ministero dell'Ambiente e della Tutela del Territorio, Direzione per la protezione della Natura, 2004)

MATT. in *Mappatura delle praterie di Posidonia oceanica e di altre fanerogame marine lungo le coste della Campania e della Calabria e delle isole minori circostanti. Relazione finale,* Vol. 2, 240 pp. (Roma: Ministero dell'Ambiente e della Tutela del Territorio, Direzione per la protezione della Natura, 2004).

Matta, E. *et al.* Mapping *Posidonia* meadow from high spatial resolution images in the gulf of Oristano (Italy) in *Geoscience and Remote Sensing Symposium (IGARSS)* (IEEE International, 2014).

Mayot, N., Boudouresque, C. F. & Leriche, A. Unexpected response of the seagrass *Posidonia oceanica* to a warm-water episode in the North Western Mediterranean Sea. *Compt. Rend. Biol.* **328**, 291–296 (2005).

MedSudMed. Report of the MedSudMed Expert Consultation on Marine Protected Areas and Fisheries Management, Salammbô, Tunisia, 14-16 April 2003. GCP/RER/010/ITA/MSM-TD-03. *MedSudMed Tech. Doc.* **3**, 1-100 (2007).

Meinesz, A. & Laurent, R. Cartes de la limite inférieure de l'herbier de *Posidonia oceanica* dans les Alpes Maritimes (France). *Ann. I. Oceanogr. Paris* **56**, 45-54 (1980).

Meinesz, A. & Laurent, R. Cartographie et état de la limite inférieure de l'herbier de *Posidonia oceanica* dans les Alpes-maritimes (France). *Bot. Mar.* **21,** 513-526 (1978).

Meinesz, A. *et al.* Impact de l'aménagement du domaine maritime sur l'étage infralittoral des Bouches du Rhône (France, Méditerranée Occidentale). *Vie et Milieu* **32,** 115-124 (1982).

Meinesz, A. *et al.* Impact de l'aménagement du domaine maritime sur l'étage infralittoral du Var, France (Mediterranée occidentale). *Ann. Inst. oceanogr. Paris* **57,** 65-77 (1981).

Meinesz, A. *et al. Posidonia oceanica* in the Marmara Sea. *Aquat. Bot.* **90,** 18-22 (2009).

Misfud, C. *et al.* The distribution and state of health of *Posidonia oceanica* (L.) Delile meadows along the maltese territorial waters. *Biol. Mar. Mediterr.* **13**, 255-261 (2006).

Montefalcone, M. *et al.* BACI design reveals the decline of the seagrass *Posidonia oceanica* induced by anchoring. *Mar. Pollut. Bull.* **56**, 1637-1645 (2008).

Montefalcone, M., Albertelli, G., Morri, C. & Bianchi, C. N. Urban seagrass: status of *Posidonia* oceanica facing the Genoa city waterfront (Italy) and implications for management. *Mar. Pollut. Bull.* **54**, 206–213 (2007).

Montefalcone, M., Albertelli, G., Morri, C., Parravicini, V. & Bianchi, C. N. Legal protection is not enough: *Posidonia oceanica* meadows in marine protected areas are not healthier than those in unprotected areas of the northwest Mediterranean Sea. *Mar. Pollut. Bull.* **58**, 515-519 (2009).

Moreno, D. & Guirado, J. Nuevos datos sobre la distribución de las fanerógamas marinas en las provincias de Almeria y Granada (SE España). *Acta Botanica Malacitana* **28,** 105-120 (2003).

Moreno, D. *et al.* Assessment of the conservation status of seagrass (*Posidonia oceanica*) meadows: implications for monitoring strategy and the decision-making process. *Biol. Conserv.* **102**, 325-332 (2001).

Moreno, D. *et al.* in *Valoración del impacto de los vertidos hídricos industriales en el litoral: aproximación metodológica al estudio de la pradera de Posidonia oceanica (L.) Delile* (Universidad de Almeria, 1999).

Mostafa, H.M. Preliminary ecological survey of seagrass beds at Marsa Matrouh, west of Alexandria-Egypt. *Biol. Mar. Mediterr.* **13**, 72-76 (2006).

NAUTILUS Scarl in *Mappatura delle praterie di Posidonia oceanica lungo le coste della Sardegna e delle piccole isole circostanti. Relazione finale*, Vol. 1, 203 pp. (NAUTILUS Scarl, 2002).

NAUTILUS Scarl in *Mappatura delle praterie di Posidonia oceanica lungo le coste della Sardegna e delle piccole isole circostanti. Relazione finale*, Vol. 1, 321 pp. (NAUTILUS Scarl, 2002).

Orth, R. & Dennison, W. *Global Seagrass Trajectories Database Compiled October. National Center for Ecological Analysis and Synthesis* (2006). Available at: https://knb.ecoinformatics.org/knb/metacat/olyarnik.3.6/nceas (Accessed: 5th March 2015)

Paillard, M. *et al.* in *Cartographie de l'herbier de Posidonie et des fonds marins environnants de Toulon a Hyères (Var, France). Reconnaissance par sonar latéral et photographie aérienne.* 36 pp. (IFREMER/GIS Posidonie, 2003).

Pasqualini, V. in *Caractérisation des peuplements et types de fonds le long du littoral corse (Méditerranée, France). PhD Thesis.* 172 pp. (Université de Corse, 1997).

Pasqualini, V., Pergent-Martini, C., Clabaut, P. & Pergent, G. Mapping of *Posidonia oceanica* using aerial photographs and side-scan sonar: application of the island of Corsica (France). *Estuar. Coast. Shelf S.* **47**, 359-367 (1998).

Peirano, A. & Bianchi, C. N. Decline of the seagrass *Posidonia oceanica* in response to environmental disturbance: a simulation-like approach off Liguria (NW Mediterranean Sea) in *The Response of Marine Organisms to their Environments* (eds Hawkins, E. & Hutchinson, S.) 87-95 (University of Southampton, 1997).

Peirano, A. *et al.* Effects of climate, invasive species and anthropogenic impacts on the growth of the seagrass *Posidonia oceanica* (L.) Delile in Liguria (NW Mediterranean Sea). *Mar. Poll. Bull.* **50,** 817-822 (2005).

Peres, J. M. & Picard, J. Causes de la raréfaction et de la disparition des herbiers de *Posidonia* oceanica sur les cotes francaises de la Méditerranée. *Aquat. Bot.* **1**, 133-139 (1975).

Pergent, G. & Pergent, C. Cartographie de l'herbier à *Posidonia oceanica* de la baie d'Urla-Iskele (Turquie). *Rapp. Comm. Int. Mer Medit.* **29**, 231-234 (1985).

Pergent, G. & Pergent, C. Cartographie de l'herbier à *Posidonia oceanica* (L.) de la baie d'Urla-Iskele (Turquie). *V. Reun. Commiss, internation. Explor. sci. Medit., Monaco* (1985).

Pergent, G. *et al.* Characterization of the benthic vegetation in the Farwà Lagoon (Libya). *J. Coastal Conserv.* **8**, 119-126 (2002).

Pergent, G. *et al.* in *Mediterranean seagrass meadows: resilience and contribution to climate change mitigation. A short summary* 40 pp. (IUCN, 2012).

Pergent, G. *et al.* Preliminary data on the impact of fish farming facilities on *Posidonia oceanica* meadows in the Mediterranean. *Oceanologica* **22,** 95-107 (1999)..

Pergent, G. *et al.* Quelques observations sur les herbiers à *Posidonia oceanica* des Pyrenées-Orientales (Méditerranée, France). *Rapp. Comm. Int. Mer. Medit.* **28,** 169-170 (1983).

Pergent, G. *et al.* Setting up the Medposidonia Programme in the Mediterranean region in *Proceedings of the Tenth International Conference on the Mediterranean Coastal Environment, Rhodes, Greece, 25-29 October 2011* (ed Ozhan, E.) 241-252 (Middle East Technical University, Ankara, 2011).

Pergent, G. *et al.* Structure of *P.oceanica* meadows in the vicinity of Ain Al-Gaza Lagoon (Libya): the "Macrotall" ecomorphosis in *Proceedings of the third Mediterranean Symposium on Marine Vegetation, France, Marseille, 27-29 March* (eds Pergent-Martini, C., El Asmi, S. & Le Ravallec, C.) 135-140 (RAC/SPA, 2007).

Pergent-Martini, C. *et al.* Impact of fish farming facilities on *Posidonia oceanica* meadows: a review. *Mar. Ecol.* 27: 310-319 (2006).

Pergent-Martini, C. *et al.* in *Mapping of coastlines of the Republic of Cyprus, Progress report.* 38 pp. (GIS Posidonie, 2013).

Pessani, D. *et al.* in *Premières données sur la distribution de Posidonia oceanica Delile autour de l'île de Saligna (îles Eoliennes, Sicilie). International Workshop on Posidonia oceanica beds* (GIS-Posidonie, 1984).

Piazzi, L. *et al.* Mappatura e monitoraggio di una prateria a *Posidonia oceanica* (L.) Delile situata a sud di Livorno (Toscana, Italia). *Inf. Bot. Italiano* **28**, 67-77 (1996).

Piazzi, L., Acuto, S. & Cinelli, F. Mapping of *Posidonia oceanica* beds around Elba Island (western Mediterranean) with integration of direct and indirect methods. *Oceanol. Acta* **23**, 339-346 (2000).

Piazzi, L., Acuto, S., Papi, I., Pardi, G. & Cinelli, F. Mappatura delle praterie a fanerogame marine della Toscana. *Biol. Mar. Mediterr.* **7**, 594-596 (2000).

Picard, J. & Bourcier M. Evolution sous influences humaines des peuplements benthiques des parages de La Ciotat entre 1954 et 1972. *Tethys* **7,** 213-222 (1975).

Picard, J. Impact sur le benthos marin de quelques grands types de nuissances liées à l'évolution de complexes urbains et industriels de la province occidentale. *Oceanis* **4,** 214-251 (1978).

Pititto, F. *et al.* Cartografia e protezione delle praterie di *Posidonia oceanica* lungo la costa albanese. *Biol. Mar. Mediterr.* **16,** 324-325 (2009).

PNUE-PAM-CAR PAP. *Programme d'aménagement côtièr (PAC) de la "Zone côtière algéroise": rapport final intégré* 237 pp. (UNEP/MAP, 2006).

Procaccini, G. *et al.* The seagrasses of the Western Mediterranean in *World Atlas of Seagrasses* (eds Green, E. P. & Short, F. T.) 48-58 (University of California Press, 2003).

Rais, C., Pergent, G., Dupuy de la Grandrive, R. & Djellouli, A. *Rapport sur le projet MedPosidonia* 137 pp. (UNEP, 2009).

Ramos Esplá, A. A. Cartografía de la pradera superficial de *Posidonia oceanica* en la bahía de Alicante (SE, España). *Intern. Workshop on Posidonia Beds* **1**, 57-61 (1984).

Renom, P. *et al.* in *Red de vigilancia de las praderas de fanerógamas marinas en Cataluña.* (Escuela del Mar de Badalona - Direcció General de Pesca i Afers Marítims, 2000).

Romero, J., Martinez-Crego, B., Alcoverro, T. & Perez, M. Corrigendum of: a multivariate index based on the seagrass *Posidonia oceanica* (POMI) to assess ecological status of coastal waters under the Water Framework Directive (WFD) in marine pollution bulletin 55: 196–204. *Mar. Pollut. Bull.* **54**, 631 (2007).

Romero, J., Perez, M. & Alcoverro, T. The seagrass (*Posidonia oceanica*) meadows in the Catalan coast: past trends and present status in *Proceedings of the third Mediterranean Symposium on Marine Vegetation, France, Marseille, 27-29 March* (eds Pergent-Martini, C., El Asmi, S. & Le Ravallec, C.) 158-163 (RAC/SPA, 2007).

Ruiz, J. M. & Romero, J. Effects of disturbances caused by coastal constructions on spatial structure, growth dynamics and photosynthesis of the seagrass *Posidonia oceanica*. *Mar. Pollut. Bul.* **46**, 1523–1533 (2003).

Ruiz, J. M., Perez, M. & Romero, J. Effects of fish farm loadings on seagrass (*Posidonia oceanica*) distribution, growth and photosynthesis. *Mar. Poll. Bull.* **42,** 749-760 (2001).

Ruiz, J., Marín, A.,Calvo J. F. & Ramírez-Diaz L. Interactions between a Floodway and Coastal Constructions in Aguilas Bay (Southeastern Spain). *Ocean Coast. Manag.* **19,** 241-262 (1993).

Sánchez Poveda, M., Martín Pato, M. A. & Sanchez-Lizsao J. L. in *Un nuevo índice para caracterizar el estado de conservación de las praderas de Posidonia oceanica (L.) Delile. 12ª reunión bienal de la Real Sociedad Española de Historia Natural* (Real Sociedad Española de Historia Natural, 1996).

Sánchez-Jerez, P. *et al.* Influence of the structure of *Posidonia oceanica* meadows modified by bottom trawling on crustacean assemblages: comparison of amphipods and decapods. *Sci. Mar.* **64,** 319-326 (2000).

Sánchez-Lisazo, J. L. *et al.* The regression of *Posidonia oceanica* meadows in El Campello (Spain). *Rapp. Comm. int. Mer. Médit.* (1990).

Semroud, R., Varlaque, R., Crouzet, A. & Boudouresque, C. F. On a broad-living form of the seagrass *P. oceanica* from Algiers. *Aquat. Bot.* **43**, 181-198 (1992).

Short, F. T. & Wyllie-Echeverria, S. Natural and human induced disturbance of seagrasses. *Environ. Conserv.* **23**, 17–27 (1996).

Short, F. T. *et al.* Extinction risk assessment of the World's seagrass species. *Biol. Conserv.* **144**, 1961–1971 (2011).

Short, F. T., Coles, R. G. & Pergent-Martini, C. Global seagrass distribution in *Seagrass Research Methods* (eds Short, F. T. & Coles, R. G.) 5-30 (Elsevier, 2001).

Silvestre, F., Iacono, G., Tiberti, L. & Buia, C. M. *Posidonia oceanica* warehousing. *Biol. Mar. Mediterr.* **13**, 277–281 (2006).

SINPOS. in *Mappatura delle praterie di Posidonia oceanica lungo le coste della Sicilia e delle isole minori circostanti–Relazione finale, contratto reperterio no. 85 del 1999*, 644 pp. (Italia: Ministero dell'Ambiente, Ispettorato Centrale per la Difesa del mare, 2001).

SNAMPROGETTI. in *Mappatura delle praterie di Posidonia oceanica lungo le coste delle regioni Liguria, Toscana, Lazio, Basilicata e Puglia. Relazione per il Ministero della Marina Mercantile*, 129 pp. (Italia: Ministero dell'Ambiente, Ispettorato Centrale per la Difesa del Mare, 1991).

Solis-Weiss, V., Aleffi, F., Bettoso, N. & Rossin, P. Gli indicatori biologici nel benthos del Golfo di Trieste. *Biol. Mar. Mediterr.* **11**, 351-354 (2004).

Torres, J. *et al.* in *Distribution and preliminary evaluation of the state of the Posidonia oceanica meadows on the coasts of Alicante (Spain, Western Mediterranean).* (Commission internationale pour l'exploration scientifique de la mer Méditerranée, 1990).

Turk, R. & Lipej, L. Research on seagrasses of the Slovenian coast (Northern Adriatic), state of the art. *Biol. Mar. Mediterr.* **13**, 282-286 (2006).

Turk, R. Main phenological characteristics of *Posidonia oceanica* (L.) Delile in the gulf of Koper (Gulf of Trieste), North Adriatic. *Biol Mar. Medit.* **7,** 131-142 (2000).

Tutin, T. G., Heywood, V. H., Burges, N. A. & Valentine, D. H. in *Flora Europaea Series,* Vol. 5, 476 pp. (Cambridge University Press, 1964).

UNEP-MAP RAC/SPA in *The Mediterranean Sea biodiversity: state of the ecosystems, pressures, impacts and future priorities*, 100 pp. (RAC/SPA, 2010).

UNEP-MAP RAC/SPA. in *Action Plan for the conservation of marine vegetation in the Mediterranean Sea,* 47 pp. (RAC/SPA, 1999).

UNEP-MAP RAC/SPA. in *Integrated coastal area management in Cyprus: biodiversity concerns on the Coastal Area Management Programme of Cyprus,* 69 pp. (RAC/SPA, 2007).

UNEP-MAP RAC/SPA. in *State of knowledge on the geographical distribution of marine Magnoliophyta meadows in the Mediterranean* 374 pp. (UNEP, 2009).

UNEP-WCMC & Short, F. T., Global distribution of seagrasses (version 2). Updated version of the data layer used in Green and Short (2003). (2005) Available at: http://data.unep-wcmc.org/datasets/. (Accessed: 25th February 2015)

Vassallo, P. *et al.* The value of the seagrass *Posidonia oceanica*: a natural capital assessment. *Mar. Pollut. Bull.* **75**, 157-167 (2013).

Vega Fernández, T., Milazzo, M., Badalamenti, F. & Danna, G. Comparison of the fish assemblages associated with *Posidonia oceanica* after the partial loss and consequent fragmentation of the meadow. *Estuar. Coast. Shelf S.* **65,** 645-653 (2005).

Zavodnik, N. & Jaklin, A. Long-term changes in the northern Adriatic marine phanerogam beds. *Rapp. Comm. int. Mer Médit.* **32,** 15 (1990).

Zupo, V. *et al.* Temporal variations in the spatial distribution of shoot density in a *Posidonia oceanica* meadow and patterns of genetic diversity. *Mar. Ecol.* **27,** 328-338 (2006).

Websites (Accessed: 5th March 2015)

Mediterranean Sensitive Habitats (MediSeH) Project
http://mareaproject.net/contracts/2/overview/

General Bathymetric Chart of the Oceans (GEBCO) Project
http://www.gebco.net

Posidonia oceanica Monitoring Network in Balearic Islands, LIFE Project
http://lifeposidonia.caib.es

Posidonia oceanica Monitoring Network in Andalusia, LIFE Project
http://www.lifeposidoniandalucia.es

Posidonia oceanica Monitoring Network in Cataluna
http://www20.gencat.cat/portal/site/DAR

Posidonia oceanica Monitoring Network in Comunidad Valenciana

http://www.ecologialitoral.com

Junta de Andalucia, Cosejeria de Medio Ambiente y Ordenacion del Territorio
http://www.juntadeandalucia.es/medioambiente/rediam/descargas_informacion_ambiental

GIS Posidonie, *Posidonia* Monitoring Network in the NW Mediterranean Sea
http://www.mio.univ-amu.fr/gisposidonie/

Water quality and Seagrass mapping, an opportunity for Greek Aquaculture – ESA aquaculture consultation
http://due.esrin.esa.int/files/m253/RS08_pkm027-529-1.0_ESA_aquaculture_consultation.pdf

Monitoring of the impact of coastal development projects on *Posidonia ocedanica* meadows
http://www.ecoserv.com.mt/Current%20Projects.htm

European Seagrass network: European Cooperation in Science and Technology (COST)
http://www.cost.eu/COST_Actions/essem/Actions/ES0906

Global Seagrass Monitoring Network (SegrassNet)
http://www.seagrassnet.org/

World Seagrass Association
http://wsa.seagrassonline.org/

Join Nature Conservation Committee (JNCC):
EMODnet – EUSeaMap, Pilot portal for broadscale modelled seabed habitats
http://www.jncc.gov.uk/page-5040

Regional Activity Center for Specially Protected Areas (RAC/SPA)
http://www.rac-spa.org/

NATURA 2000 Network
http://natura2000.eea.europa.eu/#

European Environmental Agency (EEA)
http://www.eea.europa.eu/data-and-maps/figures/distribution-of-the-marine-angiosperm-posidonia-oceanica-and-zostera-sp-in-the-mediterranean

IFREMER Sextant
http://sextant.ifremer.fr/fr/

Malta Environment and Planning Authority (MEPA) - Geoportal MapSever
http://www.mepa.org.mt/mepa-mapserver
